# Multidisciplinary High-Fidelity Simulation Training for the Management of Laryngospasm Following General and Local Anesthetic Procedures

**DOI:** 10.7759/cureus.95041

**Published:** 2025-10-21

**Authors:** Suliman Ali, Yadsan Devabalan, Chuanyu Gao, Rohan Bidaye

**Affiliations:** 1 Department of Otolaryngology, Northampton General Hospital NHS Trust, Northampton, GBR

**Keywords:** airway management, laryngospasm, multidisciplinary teamwork, patient safety, simulation-based training

## Abstract

Introduction: Laryngospasm is a potentially life-threatening airway emergency characterized by sudden, involuntary contraction of the laryngeal muscles, leading to partial or complete airway obstruction. Effective management of laryngospasm hinges on prompt recognition and appropriate intervention. The increase in laryngeal procedures performed under local anesthesia in outpatient settings necessitates that all involved multidisciplinary healthcare professionals be proficient in recognizing and managing laryngospasm promptly.

Methods: A simulation-based training day was designed to provide a structured, multidisciplinary approach to the recognition and management of laryngospasm through both theoretical learning and hands-on practical experience during two high-fidelity simulation scenarios, using advanced manikins and a realistic clinical environment to replicate real patient responses and team dynamics (local anesthetic and general anesthetic). Each scenario was followed by a structured debriefing session where participants received immediate feedback from faculty members. Pre- and post-course feedback forms were collected to assess changes in knowledge, confidence, and perceived competence, each considered a primary outcome representing complementary aspects of learning targeted by the training.

Results: Around 19 pre- and post-questionnaires were evaluated. Roles varied from resident doctors, ENT consultants, healthcare assistants, nurses, CNS, and speech and language therapists. All participants completed both simulation scenarios, and feedback was collected after the full training day. Overall, there was a statistically significant improvement (p<0.05) in participants’ confidence and perceived competence in recognizing and managing laryngospasm.

Conclusions: This study suggests that a multidisciplinary high-fidelity simulation workshop can enhance confidence and perceived competence in managing laryngospasm across both local and general anesthetic scenarios. Although knowledge gains were not statistically significant, the training’s greatest value lies in fostering non-technical skills such as communication, leadership, and teamwork, which underpin effective airway crisis management and patient safety. Future sessions incorporating larger cohorts, objective assessments, and anesthetist participation may further strengthen the educational and clinical impact of this multidisciplinary approach.

## Introduction

A potentially life-threatening airway emergency, laryngospasm is characterized by sudden, involuntary contraction of the laryngeal muscles, leading to partial or complete airway obstruction. This can be life-threatening if not appropriately managed and therefore requires prompt recognition and immediate treatment [1]. Clinically, laryngospasm presents with paroxysmal coughing, inspiratory stridor, and respiratory distress and is often accompanied by signs of increasing respiratory effort such as chest retractions, paradoxical breathing, and progressive hypoxemia [2]. While typically self-limiting, severe cases may result in significant complications such as negative pressure pulmonary edema, bradycardia, hypoxemia, and, in extreme cases, cardiac arrest [3].

Laryngospasm occurs in various clinical settings and can be triggered by multiple factors, many of which are encountered in the perioperative period. These can include the depth of anesthesia being insufficient or volatile anesthetic agents being used, such as desflurane [4]. Surgical interventions involving the airway, such as bronchoscopy, have the highest incidence, as well as tonsillectomy and adenoidectomy, being well-documented risks [5,6]. Furthermore, patient-specific factors such as laryngopharyngeal reflux disease, gastroesophageal reflux disease (GORD), upper respiratory tract infections, and irritants such as blood or tobacco smoke exposure can provoke laryngospasm by irritating the laryngeal tissues and stimulating the airway reflex responses in the laryngeal mucosa [7].

Effective management of laryngospasm hinges on prompt recognition and appropriate intervention. The initial approach involves calling for help, the immediate cessation of any airway irritants, such as suctioning secretions, blood, or foreign material that may be exacerbating the laryngospasm and airway maneuvers (chin lift and jaw thrust) to help lift supraglottic structures away from the false vocal cords, followed by the placement of oropharyngeal or nasopharyngeal airways to ensure patency of the supraglottic airway, and finally the application of continuous positive airway pressure (CPAP) [8]. In cases where these measures are ineffective, pharmacological interventions such as the administration of a small dose of propofol or a neuromuscular blocking agent like succinylcholine may be required to induce laryngeal muscle relaxation and subsequently facilitate airway control [9].

Managing laryngospasm can be alarming for staff, given the need for a prompt airway intervention due to the potentially life-threatening nature of the condition. While anesthetists are trained in laryngospasm airway management simulation as part of their formal education, the incidence of laryngospasm in non-operating room environments, where no anesthetist is present, is on the rise [10]. The increase in laryngeal procedures performed under local anesthesia in outpatient settings necessitates that all involved multidisciplinary healthcare professionals, including ENT surgeons, nurses, speech and language therapists, and other clinical staff, be proficient in recognizing and managing laryngospasm promptly. As a shared responsibility, a well-coordinated and assured team response is critical to ensuring patient safety, enabling timely initiation of management without heavy reliance on senior staff being immediately present, and preventing adverse outcomes.

Simulation-based training has emerged as an effective educational tool in medical training, offering a risk-free environment in which healthcare professionals can develop and refine their technical and non-technical skills [11]. Through scenario-based learning, simulation training allows for repeated exposure to critical situations, promoting rapid decision-making and enhancing teamwork under pressure [12]. Multidisciplinary simulation training is particularly beneficial in crisis management, where interdisciplinary coordination is essential to improving patient safety [13].

Recognizing the urgent need for a structured, multidisciplinary approach to laryngospasm management, a dedicated simulation-based training day was organized. This aimed to provide a high-fidelity, risk-free environment for participants to refine their airway management skills, reinforcing best practices in recognizing and managing laryngospasm across various clinical settings, ultimately improving patient safety and clinical outcomes in settings where laryngospasm is an ever-present risk. By evaluating participant feedback and knowledge acquisition before and after the training, this study seeks to demonstrate the benefits of simulation-based education in improving preparedness for laryngospasm across various healthcare disciplines.

## Materials and methods

The study was conducted at Kettering General Hospital, Kettering, United Kingdom. The simulation-based training day was designed to provide a structured, multidisciplinary approach to the recognition and management of laryngospasm through both theoretical learning and hands-on practical experience. The training was delivered in a dedicated simulation suite equipped with high-fidelity airway mannequins and standard clinical equipment to replicate real-world conditions. The day began with a didactic lecture delivered by a consultant laryngologist, which provided participants with a comprehensive overview of the pathophysiology, prevention, and stepwise management of laryngospasm. The lecture included discussion of common triggers, airway maneuvers, and pharmacological interventions, as well as a review of multidisciplinary team roles during airway emergencies. This ensures participants have a strong theoretical foundation that they can apply to their two simulation-based high-fidelity case scenarios with mannequins later in the day.

**Figure 1:**
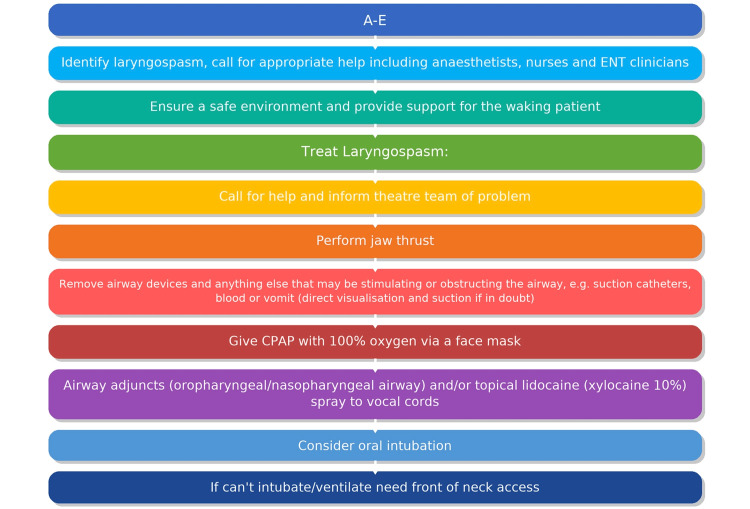
Algorithm for the identification and management of laryngospasm. A-E: airway, breathing, circulation, disability, and exposure; CPAP: continuous positive airway pressure Image credit: Suliman Ali

Following the lecture, all attendees participated in two high-fidelity simulation scenarios, ensuring consistent exposure and learning across cases. The first scenario simulated laryngospasm occurring under local anesthesia, mimicking a patient developing airway obstruction post left-sided vocal cord injection medialization. Participants were required to recognize the clinical signs of laryngospasm in an awake patient, initiate appropriate airway maneuvers, call for help from the multidisciplinary team, and administer suitable interventions such as oxygen therapy and topical anesthesia. The second scenario simulated laryngospasm occurring in a pediatric patient following general anesthesia and the removal of a laryngeal mask airway (LMA) after an uneventful bilateral grommet insertion. In this case, participants practiced early identification of laryngospasm, calling for urgent anesthetic support, and employing appropriate treatment strategies such as CPAP, jaw thrust, oxygenation, and, if necessary, pharmacological intervention. Both scenarios were designed to replicate realistic clinical presentations, including progressive airway obstruction, desaturation, and patient distress, allowing participants to practice early recognition, communication, and appropriate intervention. Figure 1 shows an algorithm summarizing how to manage these cases.

The facilitators included a consultant laryngologist and two senior ENT trainees, who guided the simulations and provided real-time observation and feedback. Each scenario was followed by a structured debriefing session, conducted by the faculty using a reflective, learner-centered approach. These debriefings focused on both technical and non-technical aspects of performance, such as decision-making, communication, teamwork, and role allocation. This allowed participants to reflect on their actions and identify opportunities for improvement in a supportive environment.

A 15-item pre- and post-course questionnaire (Appendix 1) was collected to assess changes in knowledge, confidence, and perceived competence in managing laryngospasm using a five-point Likert scale. The questionnaires were collected anonymously, and all participants completed both assessments after finishing the entire training day. Statistical analysis of pre- and post-course responses was performed using the Mann-Whitney U test, as the data were non-parametric and unpaired due to the anonymous design. A p<0.05 was deemed clinically significant.

## Results

Around 19 members of the multidisciplinary team (MDT) participated in this laryngology simulation day. Table 1 displays the various roles of the participants. Table 2 demonstrates the domains assessed in the participants. Confidence improved significantly (U=12.0, p<0.05), and competence improved significantly (U=16, p<0.05). Knowledge scores did not show a statistically significant improvement (U=70, p>0.05).

**Table 1:** Roles of multidisciplinary team (MDT) participants. Data presented as n=19 (%).

Role	N (%)
Doctors	9 (47.4%)
Nurses	5 (26.3%)
Health Care Assistants	3 (15.8%)
Speech and Language Therapists	2 (10.5%)

**Table 2:** Pre- and post-course responses for domains assessed. Data presented as median. Interquartile range presented in brackets (IQR). Statistical significance was tested with the Mann-Whitney U test (p<0.05 considered statistically significant). * indicates statistically significant result. The “% Agree or Strongly Agree” column refers specifically to post-course responses, representing the proportion of participants who selected “Agree” or “Strongly Agree” on five-point Likert scale statements assessing their perceived knowledge, confidence, and competence after completing the simulation training.

Domain	Pre-course Median (IQR)	Post-course Median (IQR)	% “Agree or Strongly Agree” (Post-course)	U-value	p-value
Knowledge	3 (2–4)	4 (4–5)	73.7%	70.0	0.342
Confidence	2 (2–3)	5 (4–5)	89.5%	12.0	0.02*
Competence	2 (2–3)	4 (4–5)	84.2%	16.0	0.04*

## Discussion

The laryngospasm simulation day suggests how structured, hands-on learning can enhance both team-based crisis management and technical proficiency. The positive feedback from attendees and the noticeable significant improvement in both confidence and competence amongst varying MDT members after the simulation day reflect how effective simulation-based training is in giving the MDT the skills to handle real-world airway emergencies. This is further shown by the pre- and post-course assessments, indicating vast improvements in attendees' ability to identify laryngospasm symptoms, initiate appropriate interventions, and collaborate efficiently with the MDT.

While there was an observed increase in participants’ knowledge scores following the session, this improvement did not reach statistical significance (p=0.342). This could be attributed to the fact that many participants were senior doctors, such as registrars and consultants, whose baseline knowledge of laryngospasm management was already relatively high. Therefore, the session may not have significantly enhanced their theoretical understanding. A more detailed subgroup analysis based on role or grade might have revealed a greater knowledge gain in less experienced groups; however, this was not performed due to the limited overall sample size, which precluded robust statistical subgroup comparisons. However, the primary aim of this multidisciplinary workshop was not solely to improve knowledge but to enhance confidence and perceived competence, both of which showed statistically significant improvement. This underscores the value of simulation training in fostering practical readiness and team-based response in airway emergencies, regardless of prior knowledge levels.

Although this is the first multidisciplinary simulation training program specifically dedicated to laryngospasm, other similar programs do exist for other ENT-related emergencies. For example, during the COVID-19 pandemic, a multidisciplinary simulation training program was introduced to help healthcare teams manage patients requiring surgical tracheostomies [14]. This study is another example indicating how multidisciplinary simulation is advantageous in preparing healthcare teams for ENT emergencies. It specifically replicated the pre-, intra-, and post-operative stages, which enhanced participants' confidence, technical skills, and interdisciplinary communication, with staff members reporting an increased sense of preparedness. Furthermore, the study demonstrated statistically significant confidence gains amongst surgeons and nurses post-simulation.

Both this study and the laryngospasm simulation day suggest improvement in confidence, skill, and collaboration by recreating high-risk scenarios in a controlled environment. The multidisciplinary simulation courses also allowed a familiarity with roles, protocols, and equipment for the participants, which is vital when responding to airway emergencies. Overall, these courses highlight how simulation-based training for airway crises can standardize practices, improve efficiency, and enhance patient outcomes.

The key strength of the laryngospasm workshop is its multidisciplinary format. Teamwork amongst different allied specialties is an essential element in managing airway emergencies and was promoted on this simulation day by the attendance of ENT surgeons, nurses, speech and language therapists, and other clinical staff. This is crucial, as laryngospasm management is not solely the responsibility of anesthetists but requires teamwork across multiple specialties and proficiency in non-technical skills, such as effective communication, role allocation, leadership, and decision-making under pressure. These allow a controlled environment where communication, teamwork, and decision-making skills can be refined and practiced. This is something that is difficult to develop in traditional lecture-based training. Patient safety is ensured during laryngospasm by healthcare workers being able to share information clearly, adapt swiftly, and collaborate efficiently.

One literature review also indicates that MDT simulation has improved soft skill development, such as teamwork, communication, and leadership, ultimately reducing errors and improving patient outcomes [15]. Furthermore, an integrative review highlighted that simulation training improved overall communication among perioperative team members [16]. Similarly, another study evaluated the effectiveness of simulation-based training on multidisciplinary critical care teams managing airway and cardiac crises. The findings indicated significant improvements in overall teamwork, leadership, team coordination, and clinical management following the training intervention [17].

These studies highlight how team dynamics and stress management are critical factors in managing clinical emergencies, and these factors are touched upon in debriefing sessions after the simulation. Thus, these structured debriefing sessions on the laryngospasm simulation day fostered reflection, promoted constructive feedback, and encouraged shared learning, which all strengthened participants’ soft skills.

The laryngospasm simulation training day tried to demonstrate face and content validity by using high-fidelity mannequins and realistic clinical scenarios, as well as having participation from MDTs. This meant the simulation closely resembled actual airway emergencies as much as possible. Furthermore, the cases used in the simulation were realistic, having actual laryngospasm symptoms and mirroring real-life clinical practice by allowing appropriate interventions to be practiced. In addition, the structured debriefing sessions further reinforced the accuracy of the clinical strategies taught.

Despite its success, the workshop did have some limitations. The main one was the lack of objective clinical outcome data, making it difficult to establish construct and predictive validity. Although valuable insights were inferred from participant feedback and self-reported confidence gains, future studies could incorporate objective performance metrics. This could include measuring response times, error rates, and decision-making accuracy.

Additionally, due to the training being conducted over a single day, its effectiveness in maintaining long-term competence may be limited. One study found that participants who underwent training sessions spaced with two-day intervals demonstrated better skill acquisition and retention compared to those with massed training sessions, suggesting that regular, spaced simulation sessions are more effective for preserving skills over time [18]. Thus, periodic refresher courses can be added to strengthen the simulation day’s credibility as a reliable educational tool. This can then be assessed by conducting follow-up assessments to evaluate skill retention over time. This would offer better insight into the program’s long-term impact, thereby improving its predictive validity and ensuring the training translates into improved clinical performance and patient safety.

As this was a pilot study, the small sample size limits the statistical power and generalizability of the findings. Participation was voluntary, which may introduce selection bias, as attendees were potentially more motivated or experienced than the wider workforce. While self-assessment remains a useful and reflective educational tool, it is subjective and may not accurately represent objective competence. Although the questionnaire demonstrated face and content validity, formal reliability and construct validity testing were not undertaken. Future studies should include a larger cohort, broaden recruitment to minimize selection bias, and incorporate validated assessment tools and objective performance measures to complement self-reported outcomes. Greater participation from anesthetists would also enhance the multidisciplinary realism and clinical applicability of the training scenarios.

Finally, the absence of a control group limits the ability to attribute the observed improvements solely to the simulation intervention. Future iterations of this program should aim to include a comparator group, such as a group that receives just lecture-based teaching, to allow for stronger causal inference and a more robust evaluation of the effectiveness of simulation-based education.

## Conclusions

Overall, this pilot study suggests that a multidisciplinary high-fidelity simulation workshop can enhance confidence and perceived competence in managing laryngospasm across a range of clinical scenarios. Although knowledge gains were not statistically significant, the training’s greatest value lies in fostering non-technical skills such as communication, leadership, and teamwork, which are key components of effective airway crisis management and patient safety.

The findings provide preliminary evidence supporting the role of multidisciplinary simulation as a valuable complement to traditional teaching methods, particularly in preparing teams for airway emergencies in both local and general anesthetic settings. Future research should include larger cohorts, objective performance assessments, and longitudinal follow-up to evaluate sustained learning and clinical translation. Incorporating control groups and periodic refresher sessions would further strengthen the evidence base and ensure the lasting educational and clinical impact of this training approach.

Ultimately, this study highlights the critical importance of embedding multidisciplinary simulation into routine training for airway emergencies. By uniting diverse clinical teams in a realistic, high-stakes learning environment, such programs not only enhance technical and cognitive performance but also strengthen the collaborative culture that safeguards patient safety.

## Appendices

Appendix 1 

**Table 3:** Pre- and post-course questionnaire Participants completed the same 15-item questionnaire before and after the simulation training day. Responses were recorded on a five-point Likert scale (1 = strongly disagree, 5 = strongly agree).

Item No.	Domain	Questionnaire Statement
1	Knowledge	I understand the pathophysiology of laryngospasm.
2	Knowledge	I can identify common causes and risk factors for laryngospasm.
3	Knowledge	I can recognize early clinical signs and symptoms of laryngospasm.
4	Knowledge	I am aware of the stepwise algorithm for managing laryngospasm.
5	Knowledge	I feel I have sufficient knowledge to treat laryngospasm in patients I encounter.
6	Confidence	I feel confident in identifying laryngospasm in a clinical setting.
7	Confidence	I feel confident in initiating immediate non-pharmacologic interventions (e.g., jaw thrust, CPAP).
8	Confidence	I feel confident in escalating to relevant team members.
9	Confidence	I feel confident in coordinating with the rest of my team and delegating roles.
10	Confidence	I feel confident in explaining laryngospasm management to learners or colleagues.
11	Competence	I believe I can manage laryngospasm effectively in a simulated environment.
12	Competence	I believe I can manage laryngospasm effectively in a real patient scenario.
13	Competence	I can remain calm and follow the algorithm under stress.
14	Competence	I can troubleshoot when initial interventions fail.
15	Competence	Overall, I feel competent in managing laryngospasm.
